# Expectations and Experiences of Spanish Primiparous Women Who Decide to Breastfeed Their Infants and Strategies for Change in 2020—A Qualitative Study

**DOI:** 10.3390/nursrep12010018

**Published:** 2022-03-02

**Authors:** Antonio Hernández-Martínez, José Miguel Quirós-García, Francisco José García-Sanchez, Miguel Ángel Puebla-Martín, David Rodríguez-Almagro, Julián Rodríguez-Almagro

**Affiliations:** 1 Department of Nursing, Physiotherapy and Occupational Therapy, Ciudad Real Faculty of Nursing, University of Castilla-La Mancha, 13071 Ciudad Real, Spain; julianj.rodriguez@uclm.es; 2 Emergency Unit, Ciudad Real University Hospital, 13005 Ciudad Real, Spain; jmquirosg@sescam.jccm.es (J.M.Q.-G.); drodrigueza@sescam.jccm.es (D.R.-A.); 3 Health Department of Junta de Comunidades de Castilla la Mancha, 45071 Ciudad Real, Spain; fjgarcia@sescam.org; 4 Pain Unit, Ciudad Real University Hospital, 13005 Ciudad Real, Spain; mapuebla@sescam.jccm.es

**Keywords:** primiparous women, breastfeeding, support healthcare professionals, nurse, nursing

## Abstract

To describe the experiences and expectations of Spanish women regarding breastfeeding and the support they receive from healthcare professionals, family, and friends during the breastfeeding journey, A qualitative study using an empirical-phenomenological approach was conducted. Primiparous women that had already given birth were interviewed using a purposive and snowball sampling. In-depth interviews were conducted between 1 January and 30 April 2020. The interviews were transcribed verbatim and analysed using Haase’s adaptation of Colaizzi’s phenomenological method. We recruited 14 women who had recently become mothers. Three major themes describing experiences of infant feeding by maternal lactation were identified—institutional influences, establishing breastfeeding, and cessation of breastfeeding—as well as the following 10 categories: hospital routines, lactation concerns (amount and infant nutrition), antenatal breastfeeding decision, embarrassment to breastfeed, and normalisation of breastfeeding. Prior education and support were identified as key elements in possible breastfeeding support strategies.

## 1. Introduction

The World Health Organisation (WHO) and the United Nations Children’s Fund (UNICEF) recommend that breastfeeding is started within the first hour after birth, is continued exclusively for the first six months of life, and then maintained along with supplementary feeding until age two or older [1]. However, although breastfeeding initiation rates are high, early discontinuation is common in different countries [2,3,4].

The benefits of breastfeeding (BF), especially exclusive breastfeeding (EBF), have been widely recognised [5,6,7,8,9,10,11]. In new-borns, BF reduces the risk of hospitalisation and morbidity [6,7,8,10,11,12,13].

However, rates for EBF continue to be low worldwide—only 36% of babies under 6 months receive EBF [12,14]. Breastfeeding is generally viewed favourably among European women [15], but breastfeeding behaviours are routinely assessed in worldwide capacities, and the WHO for the European Region has the lowest rates of EBF, with less than 25% of infants exclusively breastfed within the first six months of life [16].

The United Nations Children’s Fund (UNICEF) provides country-disaggregated data on any breastfeeding, and these rates vary widely between low- and middle-income, and high-income countries [17,18,19]. Summarising global data on any breastfeeding rates, Canada stands out with an any breastfeed rate of 89% and U.S.A. with a rate of 74.4%. In Asia, the rates of Korea (88%) and Singapore (96%) stand out [17]. A great variability has been observed between European countries, highlighting the highest rates of Nordic countries, such as Sweden (98%) and Norway (95%), which contrast with countries, such as France (63%) or Ireland (55%) [17,18]. In contrast, low- and middle-income countries rates are generally high, highlighting those in Bolivia (98.2%), Niger (98.8%), Madagascar (99%), and Bhutan (99.3) [5].

At a national level, Spain does not have a breastfeeding monitoring system, which makes it difficult to collect official data on the breastfeeding situation. However, according to the latest update of the 2018 National Health Report, in 2017, 81.1% of new-borns had been fully or partially breastfed in the first 6 weeks of life [19].

In Spain, there is no official system suitable for monitoring breastfeeding [20], but data extracted from National Health Surveys show that the rate of BF in Spain (including EBF and partial BF) at six weeks has remained relatively stable since 1995, with overall figures of around 71%. BF rates at 3 and 6 months have progressively increased in the past 5 years, reaching 66.5% and 46.9% in 2011, respectively, which is an improvement but still far from the WHO recommendations and the global target for 2025 to reach at least 50% prevalence of EBF in the first 6 months of life [21]. Data on the prevalence of BF at discharge are scarce, but in a study conducted by Vila-Candel et al., the rates reached 68.2% in 2019 [22]

An increasing number of studies suggest that parents are influenced by multiple sociocultural factors that interact to guide their infant feeding decision [20]. It has been suggested that decision making on infant feeding begins before pregnancy and is finalised in the prenatal period [23].

Regarding the support of professionals in health institutions, the existence of specialized professionals who provide continuous breastfeeding support varies greatly among centres. While some have qualified personnel dedicated exclusively to these tasks, in most public centres, breastfeeding support is part of the activities carried out by professionals on a daily basis and depends on the availability of time in their working day. This means that breastfeeding support is often limited to offering a small piece of advice [23].

Women are more likely to initiate and maintain breastfeeding behaviours when encouragement by medical professionals is combined with breastfeeding support by family, friends, and partners [5]

A qualitative study of women’s experiences of group-based or individual peer support found that women preferred a group-based approach that normalised breastfeeding and provided flexibility and a greater sense of empowerment and self-control [24]. One-to-one peer coaching was perceived as more intrusive and a greater risk to self-confidence and lacked the net social and interactional gains of a group situation [24].

The aim of this study is to describe the experiences and expectations of Spanish women regarding breastfeeding and the support they receive from health professionals, family, and friends during the breastfeeding journey.

## 2. Materials and Methods

### 2.1. Study Design and Participants

We conducted a qualitative study using in-depth interviews. An empirical-phenomenological approach was used to obtain detailed descriptions of the experiences of new mothers that were breastfeeding their infants.

### 2.2. Data Collection

Participants were recruited through purposive and snowball sampling [25]. The sample size was determined using the saturation principal, which is the number of participants at which no new categories appear with new additional participants [26]. All the participants were new mothers, considering new as the period from childbirth to one year of breastfeeding.

### 2.3. Ethical Considerations

This study received the approval of the Clinical Research and Ethics Committee.

The objectives of the present study were explained to all participants, and voluntary participation and oral informed consent were obtained before each interview. The use of numbers in place of names for each participant (e.g., mother 1: M1, etc.) and eliminating any identifying information from the transcriptions ensured confidentiality. All audio recordings and transcriptions were stored with password protection on a protected computer. Throughout the study, we followed the COREQ Standards for Reporting Qualitative Research guidelines.

### 2.4. Data Analysis

All in-person in-depth interviews were conducted at a time chosen by the participant between 1 January and 30 April 2020. The interviews were recorded with the participants permission. Data for each participant were collected, including age, civil status, duration of breastfeeding, and any education they received.

All interviews began with an open question that asked, “Please tell me how breastfeeding your infant has been for you”. Subsequently, follow-up questions were used to motivate the participants to share their story, such as: “how did you feel the first day you breastfed?”, “How do you feel now?”, “What challenges have you faced with breastfeeding?”, “Do you think you were able to resolve them?”, “Did you receive help from healthcare workers?”, and “Do you think something should be changed in the healthcare system to help women breastfeed?”. Further probing questions were added, such as “Please, tell me about that?”, to improve the depth of the interview.

Data collection was conducted at the same time as the analysis of the interviews. The interviews were conducted in the homes of the women interviewed by three different researchers. The audio recordings were transcribed verbatim by two researchers and revised by a third to ensure improved accuracy. The interviews, original transcriptions, and the data analysis were conducted in Spanish. During the data analysis, all the authors agreed with the annotations of the transcribed texts. All texts were translated to English by a native translator, and backtranslated to Spanish by an independent translator to ensure the meaning was retained, and the researchers validated the final text.

The transcriptions were analysed by Haase’s adaptation of the Colaizzi method [27,28,29]. The analysis included the reading of the transcriptions various times to understand the conveyed meanings. Significant phrases were identified and we re-formulated them to validate the meanings by consensus by the WHO research team. The themes were identified and organised into clusters and categories, developing a complete description of all the themes [29].

### 2.5. Rigour

Throughout the process, the criteria for methodological rigour of credibility, dependability, confirmability, and transferability were observed [30].

Credibility was achieved by in-depth interviews followed by peer debriefing [29,30,31]. Two co-authors analysed the transcripts independently by bracketing data on preconceived ideas and strictly following the adapted Colaizzi’s method described above [29,30,31]. Next, the findings were compared and discussed by the team until consensus was achieved on themes, theme clusters, and categories [29,30,31]. Transferability was ensured by considering variations of participant characteristics and sufficient quotations collected through in-depth interviews [29,30,31]. The audit trail was maintained to ensure that all analysis steps could be traced back to original interviews [29,30,31].

All authors participated in the validation of the results. Each step of the analysis was questioned to check any alternative interpretations. The analysis was discussed until an agreement was reached.

## 3. Results

Our sample consisted of 14 new mothers (between 1 month and 1 year breastfeeding) collected between January and April 2020. Ten had tertiary level education, and four had secondary level education (Table 1). All women were still breastfeeding their babies at the time of the interview.

The interviews lasted between 25 and 65 min. Three major themes describing experiences with infant feeding by maternal lactation were identified (Table 2): “institutional influences”, “establishing breastfeeding”, and “cessation of breastfeeding”. Ten further categories were identified within these themes.

Data saturation was reached with interview number 12, and 2 more interviews were conducted to confirm theme redundance.

### 3.1. Institutional Influences

Hospitals are complex environments in which different routine proceedings are conducted daily; the maternity wards do not vary much from this for safe growth of the new-born. However, some mothers complained that the structures imposed by hospital policies interfered with their experience of breastfeeding their infants.

“The beginning was very difficult, because of the caesarean and not being able to do skin to skin due to hospital protocol.”

(M2)

“In private hospitals, they have their protocols and, sometimes it is very difficult to make them respect the wishes of a labouring woman and a new mother.”

(M4)

“In the hospital they didn’t help me, on the contrary, they forced the infant onto the breast as per their routines, and both of us cried.”

(M6)

“The healthcare professionals in the hospital gave my infant various bottles of formula without my consent while I was in the recovery room, completely interfering with breastfeeding.”

(M8)

“In the hospital and with the medicalisation of processes it was not possible for everything to go as well as it would have at home.”

(M10)

“In the hospital after the caesarean they admitted me to the ICU for 8 h without being able to see my daughter because I had high blood pressure and they pressured the father telling him that if he didn’t sign consent to give formula they would leave her without food for the whole time I would be in ICU.”

(M3)

Only one mother considered these routines as calming processes when it came to breastfeeding.

“It is a marvellous experience, and the connection with my infant, my experience in the hospital was good, everything had a protocol, and it made me feel safe.”

(M14)

Some mothers found that the physical separation from their infant owing to admission to the neonatal intensive care unit created a challenge for breastfeeding.

“At first in the hospital it was very difficult, because of the caesarean wounds and because the infant was admitted to the ICU and they separated us. They gave the infant formula in neonates, and a dummy without even asking me.”

(M3)

Other mothers were not able to be physically present with their infant due to their own health condition. This type of situation was considered challenging, above all for the initiation of breastfeeding.

“At one month, they admitted me to the hospital with a generalised infection, and following milk culture detected infection with MRSA, a super-resistant bacterium. I had to pump milk and have intravenous antibiotic therapy.”

(M13)

Some mothers reported that healthcare professionals did not encourage them to breastfeed their babies outside of the habitual feeding hours, which discouraged them and even made them consider formula.

“In the hospital where my son was born, the help to initiate breastfeeding was some posts on the ward, 0 lactation consultants, their presence on the ward should be compulsory just like doctors, nurses, nurse aides, etc.”

(M1)

“I did not receive help from the staff of the hospital where my daughter was born.”

(M7)

“In the hospital they recommended we supplement, and I pump milk to produce more.”

(M9)

“In the hospital after childbirth there was little help.”

(M11)

“In the hospital I didn´t have the needed support, there were many contradictions between the different professionals that cared for me.”

(M13)

“The lack of interest from the healthcare professionals on the hospital ward ensured no one helped me.”

(M2)

Although when they spoke specifically about the nurses, they were considered as one of the most positive influencing factors in the experience of breastfeeding, with nurses considered as calming, informed persons that provide all the support in this initial process.

“In the hospital, I received help and support from the lactation consultant nurses.”

(M14)

“In the room, the nurses always placed my son on my breast when they entered and helped me with breastfeeding.”

(M4)

“I gave birth in the hospital where I received a lot of support by nursing staff to initiate breastfeeding, correcting posture, etc.”

(M5)

“I believe that the training of staff in public hospitals is terrible and has to change for the good of our children.”

(M8)

“Some nurses in the hospital were kinder and more patient with me than others when teaching me how to breastfeed.”

(M3)

“I am convinced that I have been able to continue breastfeeding due to the help I received from the nurses in the neonate unit of the hospital.”

(M5)

“Initially I had problems because in the hospital they gave us formula because he didn´t latch well.”

(M9)

### 3.2. Establishing Breastfeeding

The first weeks after childbirth were stressful when mothers faced the challenges of breastfeeding, with problems including insufficient milk, poor latch, or physical exhaustion due to the frequent feeding of the infant.

“It was a frustrating time, difficulties facilitating breastfeeding in the hospital, lack of time and family support, problems with a tie and latch. I tried everything: supply line, syringe, skin-to-skin, hospital pumps... Finally, I couldn´t anymore and gave up. They lack support protocols in the hospital.”

(M13)

“The start was very hard due to fissures, mastitis, drops of milk... As well as the insecurities of a first-time mum, but now I enjoy it. I would like to be able to maintain it for a time, but I don’t know if I will be able to balance it with work.”

(M2)

“Frustrating and difficult due to difficulty with latch and endless feeds that meant I didn´t sleep sufficiently.”

(M7)

“Now, without pain and confident in myself, I enjoy breastfeeding, but my infant keeps asking every hour and 45 min throughout the night, and I am exhausted, I am considering stopping after 9 months as I don’t sleep.”

(M8)

Mothers worry that their babies cannot correctly latch, which is a demotivating issue and one of the principal causes of considering stopping breastfeeding as the only nourishment for their babies.

“The start was hard because of problems with the latch that caused significant fissures, for which I started using nipple shields.”

(M6)

“In the hospital they supported me a lot with breastfeeding, but I didn´t achieve a good latch, and it ruined my nipples.”

(M1)

“My experience has always been pleasant. I feel very fortunate to have had “easy” lactation without problems with latch, or of any type.”

(M7)

In this subtheme, the correct use of breast pumps comes into play, and if the nurse showed them how to use it or, on the contrary, they did not receive any teaching by any healthcare professionals in the use of this apparatus.

“I lived attached to the breast pump to save milk for them to give him when I am working. If it wasn´t for this, I would have stopped breastfeeding a while ago.”

(M2)

“I restarted work at 6 months full-time with 5 24 h on call a month, for which I used my breast pump for a lot of time, almost up to 18 months of my son’s life.”

(M4)

“He latched on the third day and bye-bye fissures..., etc. I also want to comment that when my breast was hard and tight in the hospital, they taught me to use a pump, and this also helped a lot.”

(M11)

“My infant was a guzzler, and I had little milk despite putting him to the breast frequently with good latch and position, help arrived when in the hospital they taught me to use a breast pump, and this made breastfeeding change for the better.”

(M9)

Another important subtheme is the correct supply of milk, and this is perceived by mothers as a common occurrence and the most stressful for mothers. In the hospital environment, often the establishment of breastfeeding is achieved with the achievements of both the mother and her babies: The mother, as the producer of milk, and the babies, when we consider their success, showing they are capable of obtaining sufficient human milk as measured by weight gain.

“At first it took me a while because of engorgement and fissures, but by two months he already latched well, and milk transfer was good. I weighed him every week.”

(M4)

“My experience has been good, and the infant latched well from the first moment and gained weight well.”

(M10)

“In my case, I have bad memories of breastfeeding because the infant did not finish eating well, did not latch well, and cried a lot because of hunger.”

(M6)

An indicator that an infant is obtaining sufficient nutrients through breastmilk is often the beginning of the successful establishment of breastfeeding as a fundamental pillar in the feeding of their babies, because, if the mother believes that her child is feeding well, it is due to the success with exclusive breastfeeding without the need to offer any kind of supplementation.

“My experience at first was a little traumatic for me because my daughter didn’t latch correctly because of flat nipples and her tongue tie. She ended up losing significant weight, and I had to help with formula until she recovered her weight. Once she did, I returned to exclusive breastfeeding.”

(M13)

“The start was very complicated: nipple shields, formula supplements, and finger feeds, and supply line, poor latch, 12% weight loss, tongue tie... But with the help of the professional hospital team and the support of my partner, it all paid off, and by two months I was exclusively breastfeeding without any problems.”

(M5)

### 3.3. Cessation of Breastfeeding

The cessation of breastfeeding may occur for different reasons. In our discussions regarding the main reasons, the first reason was the decision made during the antenatal period on whether to breastfeed; in second place, it was feeling embarrassed breastfeeding in public; and lastly, it was the negative attitudes by people towards mothers that breastfeed in public. The normalisation of breastfeeding is needed to avoid unnecessary abandonment of breastfeeding.

“In my family, it’s seen as totally normal and natural. In fact, during my pregnancy I was asked a lot if I was going to breastfeed, but none of those questions came from my family–they just assumed I would.”

(M12)

Women tend to choose to breastfeed during the prenatal period based on their knowledge of breastfeeding; nevertheless, the reality of breastfeeding does not always coincide with their preformed ideal, and this incongruence between their ideal and the reality can stifle the woman’s confidence in breastfeeding and push her to wean.

“Despite having read up on it, it was full of surprises. From the start, it didn´t go well enough, leading to use of nipple shields, leaving them even though I read everywhere that it wasn’t easy and shortened breastfeeding, something that didn´t happen in my case; mastitis, a lot of pain, understand and live with each of the stages and then able to honestly enjoy it. It takes a lot, and I think that it is idealised, it is not at all rosy.”

(M4)

“I think we will tend to idealise breastfeeding and that nobody is really prepared for the setbacks. Nobody tells you that maybe you should give formula during those first days to be able to rest, it won’t interfere with breastfeeding, they tell you the opposite, also the use of the dummy, but really, my experience tells me that I was able to start with mixed feeding in some feeds, use a dummy, and still exclusively breastfeed my infant for seven months. Nobody tells you that if you can’t, it doesn´t matter. You can try again later. And above all that you are doing a GOOD job.”

(M10)

Feeling embarrassed breastfeeding in public was an important sub-theme, especially the embarrassment of the women and their families and/or society in general. Some women felt they need to ask permission before nursing in front of their friends and some family members.

“With my son, I didn´t put him on the breast initially, partly because of the visitors in the hospital and I felt embarrassed to pull out my breast in front of them.”

(M1)

“It is an experience suffered and sacrificed for the mother; sometimes, you feel embarrassed breastfeeding with people around and especially at first.”

(M2)

“The insecurity and embarrassment that one may feel breastfeeding is unfair, and many mothers wean because of that, and due to misinformation, I see it constantly.”

(M13)

Some women expressed reluctance in terms of feeding their babies in public, emphasising the need to be discrete and carried out in a hidden manner, and even if recognising that breastfeeding is acceptable in public, it should be in a way that does not appear offensive to other people.

“In general, people comment that you should pick up a child a lot, nor let them sleep with us, much less breastfeed in public.”

(M2)

“Criticism always affected me, and I felt a lot of shame nursing in public.”

(M8)

“I breastfed him very occasionally because of the short time and only in private because I was told by whoever saw me not to do it, and I was embarrassed, and in the end I stopped.”

(M6)

Some women reported having felt negative attitudes and perceptions from others towards breastfeeding; whether due to breastfeeding or for the duration of breastfeeding.

“For my first son, I stopped breastfeeding at 8 months only because of social pressure.”

(M10)

“I weaned them at one year because they fell asleep nursing all the time, and, honestly, I was very tired of people telling me I had to stop breastfeeding.”

(M6)

Facilitating a sensation of it being normal for women to feed their children via breastfeeding was considered essential by all the women, both in the promotion of breastfeeding as in the initiation and duration, and, of course, the acceptance of the whole breastfeeding process by the woman.

“I breastfed everywhere. Always when my infant was hungry. I breastfed until I felt it was ok to do so, despite the opinions of those that said I should stop earlier and those that said it was early. I think that making mothers feel bad for their lactation choices is horrible.”

(M13)

“I am proud that I was able to normalise breastfeeding around me and able to help other mothers through different paths.”

(M5)

“Breastfeeding needs to be normalised. It seems to scare people to see someone breastfeeding!!”

(M14)

“A lot more information and normalisation are needed, and above all, more help in the hospital. It helps to insist that yes, you can breastfeed.”

(M1)

## 4. Discussion

The aim of this study was to explore experiences of first-time mothers with infant feeding via breastfeeding. Our findings indicate that the experiences of infant feeding were modified by institutional influences, the period of breastfeeding establishment, and by their own cessation of breastfeeding.

The results show us that the hospital environment influenced the infant and the mother’s experience of feeding her children; while some mothers felt influenced by the processes and technological environment, others felt appreciative of the role that nurses played in informing them regarding infant feeding with breastfeeding [32,33,34].

The importance of healthcare professionals as educators in the promotion of breastfeeding is recognised by some mothers and considered a part of their role [35,36] and as a professional attitude that directly affects the attitude of the mother towards breastfeeding. 

Similar to our study, some authors have found that mothers experience a change in reality once they bring their babies home, and they find themselves facing a lack of skills and information regarding lactation, such as correct latch and ability to assess if babies are correctly feeding, as well as constant worry if they are providing their infant with sufficient breastmilk [37,38].

The hospital environment places healthcare professionals as experts and mothers as beginners in breastfeeding. All the mothers in our study regarded the nurses as experts, especially in terms of breastfeeding support [39]. Additionally, achieving objectives, such as correct latch and/or exclusive breastfeeding, provide mothers with a tangible success regarding their ability to be able to exclusively breastfeed at home [40,41].

The study by Thomson and Dykes [42] describes the mismatch or “discrepancies between theoretical expectations and those incorporated into reality”. In their study, they showed how mothers expect breastfeeding to be a natural experience, as usually the decision to breastfeed is made during the antenatal period, but on the contrary, many mothers found this experience was a difficult and even painful one, as it happened to some of the mothers in our sample. In other previous studies [43,44], some mothers were surprised with the problems that arise with breastfeeding.

A large difference exists between expectations and reality. This tension reflects the problems inherent in the maternal ideal of trying to satisfy two very demanding tasks: on the one hand, to breastfeed her infant and, on the other hand, to fulfil the role of the working mother and caretaker of her home [45]. We must normalise the process of breastfeeding, so that this dilemma does not exist for mothers.

Previous studies [46,47,48] have shown that both health professionals and breastfeeding women themselves value participation and support as a process of change against the negative comments of people towards breastfeeding that can discourage women from feeding their babies by this method. However, it is necessary to help women to understand that they can challenge these comments and opinions. These negative attitudes can be combated with the confidence built from the knowledge, support, and sense of normality that health professionals can provide as experts on the subject.

The authors believe that, in addition to improving knowledge and resources that should be offered to mothers, it is essential that health institutions promote changes in care protocols that facilitate breastfeeding. Specifically, it would be highly advisable that the centres adhere to the Baby Friendly Hospital initiative to follow a process of appropriate change and based on current scientific evidence, implementing practices, such as skin to skin in all cesarean deliveries, when the situation allows, and strengthening support for those women who are especially in vulnerable situations.

The authors are surprised that the reasons for abandoning breastfeeding do not include the incorporation to work, possibly attributable to the work situation of the sample evaluated.

### Strengths and Limitations

The use of in-depth interviews allowed mothers to explore their own experiences with breastfeeding with minimal interviewer influence.

One potential limitation in this study was the time passed from hospital discharge to the interview, which ranged from 15 days to 1 year. This time difference may have resulted in different perceptions of their experiences breastfeeding, as the babies were in different stages of their development and growth. We should also recognise that this is a small study, and we cannot generalise the findings, although, in qualitative research, a small sample size allows for the full exploration of participants’ experiences and in-depth analysis [49], and they provide a snapshot that can guide future initiatives for change.

## 5. Conclusions

This study explored the experiences and expectations of Spanish women regarding breastfeeding and the support they receive from health professionals, family, and friends during the breastfeeding journey. Specifically, it explored the possible causes for stopping breastfeeding.

Previous education and support were recognised as key points for possible strategies, and it is important for health systems and key decision-makers to provide resources to the healthcare professionals that have contact with mothers who wish to breastfeed. These resources should be compulsory and centred in the education of mothers regarding the full breastfeeding process.

These improvements in education and training would support the provision of appropriate care by healthcare professionals to help mothers in theirs and their babies’ breastfeeding journey.

Providing a realistic image of breastfeeding could contribute to building a community culture of support for mothers, understanding that, for breastfeeding, the decisions made previously have longer-term influence in the maintenance and cessation of breastfeeding.

## Figures and Tables

**Table 1:** Participant characteristics.

	Age (Years)	Civil Status	Duration of Breastfeeding	Primiparous	Employment Status	Education Level
Mother 1	21	Single	1 month	Primiparous	Employeed	University
Mother 2	24	Single	15 days	Primiparous	Unemployed	University
Mother 3	23	Married	3 months	Primiparous	Unemployed	University
Mother 4	22	Single	2 months	Primiparous	Unemployed	Secondary
Mother 5	30	Married	6 months	Primiparous	Employeed	University
Mother 6	21	Married	1 year	Primiparous	Unemployed	University
Mother 7	24	Married	7 months	Primiparous	Employeed	University
Mother 8	23	Married	5 months	Primiparous	Unemployed	University
Mother 9	35	Married	4 months	Primiparous	Employeed	Secondary
Mother 10	26	Single	8 months	Primiparous	Employeed	Secondary
Mother 11	22	Married	2 months	Primiparous	Unemployed	Secondary
Mother 12	23	Married	4 months	Primiparous	Unemployed	University
Mother 13	21	Single	3 months	Primiparous	Unemployed	University
Mother 14	22	Married	5 months	Primiparous	Unemployed	University

**Table 2:** Theme clusters and theme categories.

**1. Institutional influences**
a. Hospital routines
b. Separation of babies
c. Encouragement of breastfeeding by healthcare professionals
**2. Establishing breastfeeding**
a. Worry about correct latch
b. Sufficient human milk
c. Correct nutrition through exclusive breastfeeding
**3. Cessation of breastfeeding**
a. The choice to breastfeed during the antenatal period
b. Overcome embarrassment
c. Negative attitudes of other people
d. Normalisation of breastfeeding

## Data Availability

All in-person in-depth interviews were conducted at a time chosen by the participant between 1 January and 30 April 2020. The interviews were recorded with the participants permission. Data for each participant were collected, including age, civil status, duration of breastfeeding, and any education they had received.
